# The relationship between childhood emotional neglect experience and depressive symptoms and prefrontal resting functional connections in college students: The mediating role of reappraisal strategy

**DOI:** 10.3389/fnbeh.2023.927389

**Published:** 2023-02-22

**Authors:** Bin Xu, Shilin Wei, Xiaojuan Yin, Xiaokang Jin, Shizhen Yan, Lina Jia

**Affiliations:** Faculty of Psychology, Tianjin Normal University, Tianjin, China

**Keywords:** childhood emotional neglect, reappraisal strategy, depressive symptoms, resting-state functional magnetic resonance, college student

## Abstract

Childhood emotional neglect (CEN) has a relatively high incidence rate and substantially adverse effects. Many studies have found that CEN is closely related to emotion regulation and depression symptoms. Besides, the functional activity of the prefrontal lobe may also be related to them. However, the relationships between the above variables have not been thoroughly studied. This study recruited two groups of college students, namely, those with primary CEN (neglect group) and those without childhood trauma (control group), to explore the relationships among CEN, adulthood emotion regulation, depressive symptoms, and prefrontal resting functional connections. The methods used in this study included the Childhood Trauma Questionnaire (CTQ), Emotion Regulation Questionnaire (ERQ), Beck Depression Inventory-II (BDI-II) and resting-state functional magnetic resonance imaging (rs-fMRI). The results showed that compared with the control group, the neglect group utilized the reappraisal strategy less frequently and displayed more depressive symptoms. The prefrontal functional connections with other brain regions in the neglect group were more robust than those in the control group using less stringent multiple correction standards. Across the two groups, the functional connection strength between the right orbitofrontal gyrus and the right middle frontal gyrus significantly negatively correlated with the ERQ reappraisal score and positively correlated with the BDI-II total score; the ERQ reappraisal score wholly mediated the relationship between the functional connection strength and the BDI-II total score. It suggests that primary CEN may closely correlate with more depressive symptoms in adulthood. Furthermore, the more robust spontaneous activity of the prefrontal lobe may also be closely associated with more depressive symptoms by utilizing a reappraisal strategy less frequently.

## 1. Introduction

Childhood emotional neglect (CEN) refers to the failure to meet children's basic emotional needs, the lack of emotional response to children's pain, the inability to take into account children's social needs, and the expectation that they will deal with situations beyond their maturity or insecurity (Teicher and Samson, 2013). Several studies have identified CEN as a relatively common subtype of childhood trauma with a relatively high incidence rate and substantially adverse effects (Finkelhor et al., 2013; Dias et al., 2015; Shen et al., 2015; Maguire and Naughton, 2016; Taillieu et al., 2016).

Many studies have indicated that CEN is closely related to emotion regulation and depression symptoms. For instance, Huh et al. (2017) demonstrated that adaptive emotion regulation strategies significantly mediated the relationship between CEN and depressive symptoms. According to a study by O'Mahen et al. (2015), there was a strong correlation between the CEN score and the avoidance strategy score. This strategy significantly mediated the relationship between CEN and depressive symptoms. In addition, Wang et al. (2017) also demonstrated a significant negative correlation between CEN and reappraisal strategies in patients with depression. These studies suggest that CEN may change the tendency to utilize emotion regulation strategies in adulthood, which may be an essential reason that CEN leads to depression. Early adulthood, especially the college stage when one first leaves home life, is an important period for individuals to develop their emotion regulation ability. Exploring the relationship between CEN and college students' emotion regulation and depressive symptoms will help prevent and intervene in college students' depressive symptoms.

In addition, some studies have suggested that the CEN experience of individuals may alter not only the behaviors related to adulthood emotion regulation but also the functional activity of the brain associated with emotion regulation. Resting-state functional magnetic resonance imaging (rs-fMRI) does not require experimental tasks. Therefore, the observed individual differences in spontaneous BOLD signal fluctuations are not disturbed by experimental manipulation differences when using rs-fMRI, which reflects the inherent characteristics of a particular brain activity. In conclusion, it can be purer to explore the state-dependent brain functional differences between individuals (Tavor et al., 2016). Using rs-fMRI, Wang et al. (2014) discovered significant negative correlations between CEN and the functional connections of the bilateral thalamus and also the dorsolateral and medial prefrontal gyrus in adults with major depression. Fadel et al. (2021) found that CEN experience was correlated with decreased functional connections within the salience network and increased functional connections between the salience network and the default mode network in adults with major depression. Based on the region of interest analysis, Souza-Queiroz et al. (2016) found that CEN significantly negatively correlated with the abnormal functional connections between the left ventromedial prefrontal lobe and amygdala in adults with bipolar disorder. These studies found a close relationship between CEN and prefrontal functional connections in adults with severe depressive symptoms. The prefrontal cortex has been consistently implicated in perceiving and understanding emotional information and cognitive and attentional control (Li et al., 2020a,b). Abnormal prefrontal cortex functional activities have been reported in a large number of studies to closely correlate with difficulties in emotion regulation and emotional disorders (Wager et al., 2008; Kanske et al., 2011; Rabinak et al., 2014; Koch et al., 2016; Yang et al., 2020). In addition, because the region of interest analysis can avoid the possibility of false-negative results caused by numerous multiple comparison corrections (the brain is divided into more than 6,000 voxels) in whole-brain analyses, it may better detect small but meaningful differences (Poldrack, 2007; Price et al., 2021).

Finally, we conclude that CEN may be closely linked to the tendency to utilize emotion regulation strategies, depressive symptoms, and changes in prefrontal functional connections in adults. However, the limitations of existing literature in the screening of subjects restrict the validity of the results. As can be seen, the adults with childhood trauma in the aforementioned literature were mostly accompanied by severe physical or psychological disorders (Wang et al., 2014; Souza-Queiroz et al., 2016; Fadel et al., 2021). Moreover, even healthy control adults experienced some childhood trauma (including CEN) (Wang et al., 2014; Souza-Queiroz et al., 2016). Furthermore, some studies suggested that the relationship between CEN and emotion regulation, depressive symptoms, and brain functional activity might be specific to other childhood trauma subtypes. For example, Huh et al. (2017) demonstrated that only CEN, but not other childhood trauma subtypes, could lead to more depressive symptoms causing less frequent utilization of adaptive emotion regulation strategies. Similarly, O'Mahen et al. (2015) found that only CEN could exacerbate depressive symptoms through avoidance strategies. Dannlowski et al. (2013) discovered that just CEN, rather than other childhood trauma subtypes, was the most critical predictor of abnormal brain activity when individuals watched sad faces. The latter has been linked to many negative emotional symptoms. Thus, further research is needed to investigate whether there is a strong relationship between primary CEN experience and emotion regulation strategies, depressive symptoms, and resting state prefrontal functional connectivity abnormalities in healthy college students without psychological disorders, with interference from other traumatic experiences excluded whenever possible.

Accordingly, the present study aims to explore the relationships among primary CEN, the tendency to utilize emotion regulation strategies, depressive symptoms, and resting state prefrontal functional connections by combining Childhood Trauma Questionnaire (CTQ), Emotion Regulation Questionnaire (ERQ), Baker Depression Inventory-II (BDI-II), and rs-fMRI. First, two groups of healthy college students with primary CEN (neglect group) and no childhood trauma (control group) were screened by evaluating CTQ and clinical interviews by psychiatrists. Second, between the two groups, ERQ and BDI-II were used to investigate the differences in the tendency to utilize emotion regulation strategies and depressive symptoms, and rs-fMRI was used to investigate the differences in prefrontal functional connections. Finally, combined with the questionnaire and imaging data, the potential mediating role of emotion regulation strategies in the relationship between prefrontal functional connections and depressive symptoms was explored.

This study's hypotheses are as follows: (1) The neglect group may utilize the adaptive strategy (reappraisal) less frequently and have more depressive symptoms; (2) There may be significant differences in prefrontal functional connections with other brain regions between the two groups; and (3) The prefrontal functional connections with other brain regions may be significantly correlated with the scores of emotion regulation strategies and depressive symptoms, and emotion regulation strategies may mediate the relationship between the above functional connections and depressive symptoms.

## 2. Methods

### 2.1. Participants

Potential participants were screened by the online survey from March to June 2020. A total of 5,010 college students from four universities in Tianjin, China, participated in the survey. The content included Childhood Trauma Questionnaire (CTQ) and relevant demographic information (age, gender, student origin distribution, and whether only child). There were 4,566 questionnaires with complete details that were carefully answered, with an effective completion rate of 91.14%. After a minimum of 1 month, video interviews were conducted with potential participants identified in the initial investigation, including the retesting of CTQ, screening for mental illness, and significant physical hazards [completed jointly by two attending psychiatrists according to the DSM-IV-TR Axis I Clinical Examination Patient Edition for Disorders (SCID-I/P)].

The cutoff scores of CTQ subscales recommended by relevant studies were used as the basis for screening the two groups of participants for a formal experiment (Sáez-Francàs et al., 2015; Lu et al., 2016; Frodl et al., 2017; Kim et al., 2018; Peters et al., 2018; Wu et al., 2021). The inclusion criteria for the neglect group were as follows: emotional neglect ≥15, emotional abuse < 12, physical abuse < 9, sexual abuse < 7, and physical neglect < 9; for the control group: emotional neglect, emotional abuse, physical abuse, sexual abuse, and physical neglect all scored a minimum of 5. All the above results were consistent across two CTQ assessments for each group. Exclusion criteria for both groups were (1) any mental disorder that met the diagnostic criteria for the DSM-IV-TR axis I; (2) severe physical diseases, such as hypertension, diabetes, heart disease, thyroid disease, and basic metabolic diseases; (3) head injury with coma lasting for more than 5 min; (4) epilepsy or febrile seizures; (5) receiving or having received electroconvulsive, acupuncture, and other physical therapy; (6) being pregnant or planning to be pregnant; and (7) contraindications for MRI.

The final number of participants was 21 in the neglect group (10 male participants, 19.19 ± 0.68 years old) and 26 in the control group (13 male participants, 19.04 ± 0.87 years old). All the participants signed the informed consent and received specific remuneration after the study. The ethics committee of Tianjin Normal University approved the experimental scheme.

### 2.2. Questionnaire

#### 2.2.1. Childhood trauma

The childhood trauma of college students was assessed using the Chinese version of the Childhood Trauma Questionnaire-Short Form (CTQ-SF, Bernstein et al., 2003), translated and revised by Zhao et al. (2005). It is a retrospective self-assessment questionnaire with 28 items, assessing five factors, namely, emotional abuse, physical abuse, sexual abuse, emotional neglect, and physical neglect. Each factor contains five items; each is rated on a five-point Likert scale from 1 (never) to 5 (always). The higher the subscale scores and total score are, the more serious the individual's childhood trauma is. The Chinese version was tested on 819 high school students, and the reliability and validity were good (Zhao et al., 2005). In this study, Cronbach's α coefficient of the total scale was 0.80.

#### 2.2.2. Emotion regulation strategies

The emotion regulation strategies of college students were assessed using the Chinese version of the Emotion Regulation Questionnaire (ERQ, Gross and John, 2003), translated and revised by Wang et al. (2007). It is a retrospective self-assessment questionnaire with 10 items, assessing two factors: reappraisal and suppression. The reappraisal factor contains six items and the suppression factor contains four items; each is rated on a seven-point Likert scale from 1 (strongly disagree) to 7 (strongly agree). The higher each subscale score is, the more likely the individual will utilize this strategy. The Chinese version was tested on 1,163 college students, and the reliability and validity were good (Wang et al., 2007). In this study, Cronbach's α coefficient of the reappraisal was 0.86 and the suppression was 0.71.

#### 2.2.3. Depressive symptoms

The depressive symptoms of college students were assessed using the Chinese version of the Beck Depression Inventory-II (BDI-II, Beck et al., 1996), translated and revised by Yang et al. (2014). It is a retrospective self-assessment questionnaire with 21 items, assessing two factors, namely, cognitive-affective and somatic. The cognitive-affective factor contains 16 items, and the somatic factor contains five items; each is rated on a four-point Likert scale from 0 (no symptoms) to 4 (pronounced symptoms). The higher the subscales scores and total score are, the more serious the individual's depressive symptoms are. The Chinese version was tested on 2,797 college students, and the reliability and validity were good (Wang et al., 2018). In this study, Cronbach's α coefficient of the total scale was 0.90.

#### 2.2.4. Socioeconomic status in childhood

Previous studies have found that childhood socioeconomic status represented by family economic status, education level, and work status of the father and mother may be associated with changes in adulthood brain functional activities (Ly et al., 2011; Neville et al., 2013; Muscatell, 2018). Therefore, parent's education level and work status and also the subjective perceived family economic status during childhood were additionally collected as covariates in the following analysis of brain function to exclude the interference of the differences between groups in childhood socioeconomic status on the results. According to the study of Luo and Waite (2005), parents' education level was divided into six dimensions: 1 = none, 2 = primary school, 3 = junior middle school, 4 = high school or technical secondary school, 5 = university or junior college, and 6 = master's degree or above. Parents' work status was divided into seven dimensions: 1 = none, 2 = education system, 3 = health system, 4 = state-owned enterprises, 5 = government departments, 6 = private enterprises, and 7 = others. The subjective perceived family economic status was divided into three dimensions: 1 = poor, 2= medium, and 3 = rich.

### 2.3. Resting-state fMRI data collection and preprocessing

#### 2.3.1. Data collection

All the rs-fMRI data were collected at Tianjin Normal University. ERQ and BDI-II were completed before fMRI scanning using the Siemens MAGNETOM Prisma 3.0T Scanner and 64-channel head coil. The participants were supine and we placed a sponge pad inside the coil to fix their heads. They were asked to keep their heads and bodies still and their eyes focused on the cross on the screen without systematic thinking. For the resting state functional imaging, ABI1_bold_rest sequence and sagittal scanning were used, TR = 2,000 ms, TE = 30 ms, FA = 90°, FOV = 224 × 224 mm, matrix = 112 × 112, voxel size = 2 × 2 × 2 mm, slices = 75, slice thickness = 2 mm, no interval, time points = 240, and acceleration factor = 3. The acquisition time was 495 s. For the whole brain structural imaging, ABI1_t1iso_mprage sequence and sagittal scanning were used; TR = 2,530 ms, TE = 2.98 ms, FA = 7°, FOV = 256 × 256 mm, matrix = 256 × 256, voxel size = 1 × 1 × 1 mm, slices = 192, slice thickness = 1 mm, no interval. The acquisition time was 363 s.

#### 2.3.2. Data preprocessing

DPABI V4.3 (http://rfmri.org/dpabi) was used for data preprocessing. The steps included are as follows: (1) The images were retained in the NIFTI format; (2) The first 10 time points were deleted; (3) Slice-time correction, considering that rs-fMRI adopted the sequence with acceleration factor = 3, MATLAB R2015b (MathWorks, http://www.mathworks.com/) was used to find the reference time point of each participant, then SPM12 (http://www.fil.ion.ucl.ac.uk/spm) was used to conduct slice-time correction according to the reference time point; (4) Motion correction, the data that translation exceeds 1.5 mm and rotation exceeds 1.5°were deleted (two in the neglect group and one in the control group, see Appendix 1 for details); (5) Normalization, DARTEL was used to normalize functional images into Montreal Neurological Institute (MNI) space: the structural images of each participant were registered into the average functional images, and then the structural images were divided into gray matter, white matter, and cerebrospinal fluid, so a matrix was generated; finally, the functional images were normalized to the MNI standard space by using the matrix generated when the structural images were segmented with resampling voxel size = 2 × 2 × 2 mm; and (6) spatial smoothing (FWHM = 6 × 6 × 6 mm).

## 3. Statistical analysis

### 3.1. Demographic and questionnaire data analysis

SPSS 25.0 (IBM Corporation, Armonk, NY, USA) was used to conduct an independent sample *T*-test for the age of the two groups (*p* < 0.05) and a chi-square test for other demographic variables (*p* < 0.05). An independent sample *t*-test was conducted for the CTQ subscales scores, BDI-II total score, and ERQ reappraisal and suppression scores (*p* < 0.05).

### 3.2. Functional connection analysis of the prefrontal lobe and its correlation and mediation analysis with questionnaire data

#### 3.2.1. Extracting PFC subregions as ROI

According to the Brainnetome Atlas (BN Atlas) developed by the Brainnetome Center, Institute of Automation, Chinese Academy of Sciences (http://atlas.brainnetome.org, see Appendix 2 for details), we marked all the 68 subregions of PFC (BN1-68). After that, REST V1.8 (http://www.restfmri.net/forum) was used to extract binary masks for these subregions of ROI.

#### 3.2.2. Voxel-wise functional connection analysis

(1) After smoothing, the data were used to extract and remove covariates (including linear drift trend, Friston 24 head parameters, cerebrospinal fluid, and white matter), and then band-pass was filtered (0.01~0.1 Hz). ROIs were the aforementioned 68 subregions of PFC extracted from the BN Atlas. The average time series of all the ROIs of the two groups were extracted, their linear correlation coefficients with the voxels of the whole brain were calculated, and then Fisher-z transformation to obtain the zFC statistical map was conducted. (2) A total of 10 items of FD Jenkinson head movement parameters, age, gender, whether only child, student origin distribution, education level, work status of parents, and subjective perceived family economic status in childhood were taken as covariates (the same below), and two-sample *t*-test was conducted on the zFC statistical map of each ROI of the two groups using SPM12. Two methods, “family wise error rate” (FWE, *p* < 0.05) and “false discovery rate” (FDR, *p* < 0.05), were used for multiple corrections as the first and second options. If the results cannot pass the above multiple corrections, according to previous studies (Petersen et al., 2017; Lin et al., 2020; Schneider et al., 2021), the method “a threshold of *p* < 0.001 (uncorrected at the voxel level) followed by an empirically determined threshold of *p* < 0.05 (FWE at the extent level)” was used as the third option. All the clusters with significant differences between the two groups were labeled according to the BN Atlas and compared with AAL (Anatomical Automatic Labeling) using xjView. (3) Taking the central coordinate of the above clusters with significant differences between the two groups as the center of the circle and 3 mm as the radius, DPABI V4.3 was used to extract the zFC values of the clusters. The correlation analysis was conducted with the questionnaire data (the ERQ reappraisal and suppression scores, and the BDI-II total score), controlling all covariates (Bonferroni correction). (4) If some zFC values were significantly correlated with the ERQ reappraisal or suppression score and BDI-II total score simultaneously, the BDI-II total score was taken as the dependent variable and the zFC values and ERQ reappraisal or suppression scores were taken as the independent and mediating variables, respectively; in turn, a mediation analysis was conducted controlling all covariates.

## 4. Results

### 4.1. Differences in demographic and questionnaire data between the two groups

#### 4.1.1. Differences in demographic data between the two groups

There was no significant difference between the two groups regarding age, gender, whether only child, student origin distribution, parents' education level, and work status in childhood (*p* > 0.05). However, there were significant differences in subjective perceived family economic status in childhood (*p* = 0.005) (see Appendix 3 for details).

#### 4.1.2. Differences in questionnaire data between the two groups

As shown in Table 1, all CTQ subscales scores and the BDI-II total score in the neglect group were significantly higher than those in the control group (*p* < 0.001), and the ERQ reappraisal score was significantly lower than that in the control group (*p* < 0.001). There was no significant difference in the ERQ suppression score between the two groups (*p* > 0.05).

**Table 1:** Differences in questionnaire data between the two groups (*n* = 47).

**Variables**	**Neglect group (*****n*** = **21)**		**Control group (*****n*** = **26)**		**Cohen's d**	***t***	***p***
	***n***	***M* ±*SD***	***n***	***M* ±*SD***			
CTQ:CEN	21	16.00 ± 1.22	26	5.00 ± 0.00	12.751	45.918	< 0.001
CTQ:emotional abuse		8.10 ± 2.43		5.00 ± 0.00	1.804	6.520	< 0.001
CTQ:physical abuse		6.05 ± 1.40		5.00 ± 0.00	1.061	3.838	< 0.001
CTQ:sexual abuse		5.52 ± 0.81		5.00 ± 0.00	0.908	3.292	0.002
CTQ:physical neglect		7.76 ± 1.34		5.00 ± 0.00	2.913	10.553	< 0.001
ERQ:reappraisal		27.29 ± 5.02		34.08 ± 5.47	−1.293	−4.388	< 0.001
ERQ:suppression		15.57 ± 4.65		13.85 ± 4.75	0.366	1.250	0.218
BDI-II:total		16.38 ± 7.43		4.19 ± 4.32	2.001	7.031	< 0.001

#### 4.1.3. Correlation and linear regression analysis of questionnaire data

Controlling all covariates, partial correlation analysis showed that only the ERQ reappraisal score was significantly negatively correlated with the BDI-II total score (*r* = −0.597, *p*_correction_ < 0.001). In contrast, the ERQ suppression score was not significantly correlated with the BDI-II total score (*r* = −0.032, *p*_correction_ > 0.05).

Controlling all covariates, linear regression analysis further proved that the ERQ reappraisal score was significantly negatively correlated with the BDI-II total score [Beta = −0.698, *t* = −5.401, *p* < 0.001, 95.0% confidence interval (−1.310, – −0.586)].

### 4.2. Differences in the functional connections of PFC subregions between the two groups and the correlation with questionnaire data

#### 4.2.1. Differences in the functional connections of PFC subregions between the two groups

There was no significant difference between the two groups that could pass the multiple corrections of FWE and FDR. However, some clusters with significant differences between the two groups could pass the multiple corrections of the third method [*p* < 0.001 (uncorrected at the voxel level) followed by *p* < 0.05 (FWE at the extent level)]. In addition, the results indicated that BN7 (with BN179 and BN122), BN40 (with BN12 and BN51), BN46 (with BN22 and BN166), BN3 (with BN232), BN8 (with BN3), BN11 (with BN64), BN12 (with BN64), BN16 (with BN3), BN20 (with BN46), BN36 (with BN204), BN38 (with BN3), BN39 (with BN62), BN42 (with BN23), BN44 (with BN64), BN47 (with BN221), and BN52 (with BN204) showed more robust functional connections in the neglected group than those in the control group (see Appendix 4 for details).

#### 4.2.2. Correlation between the functional connections of PFC subregions and questionnaire data

**Figure 1:**
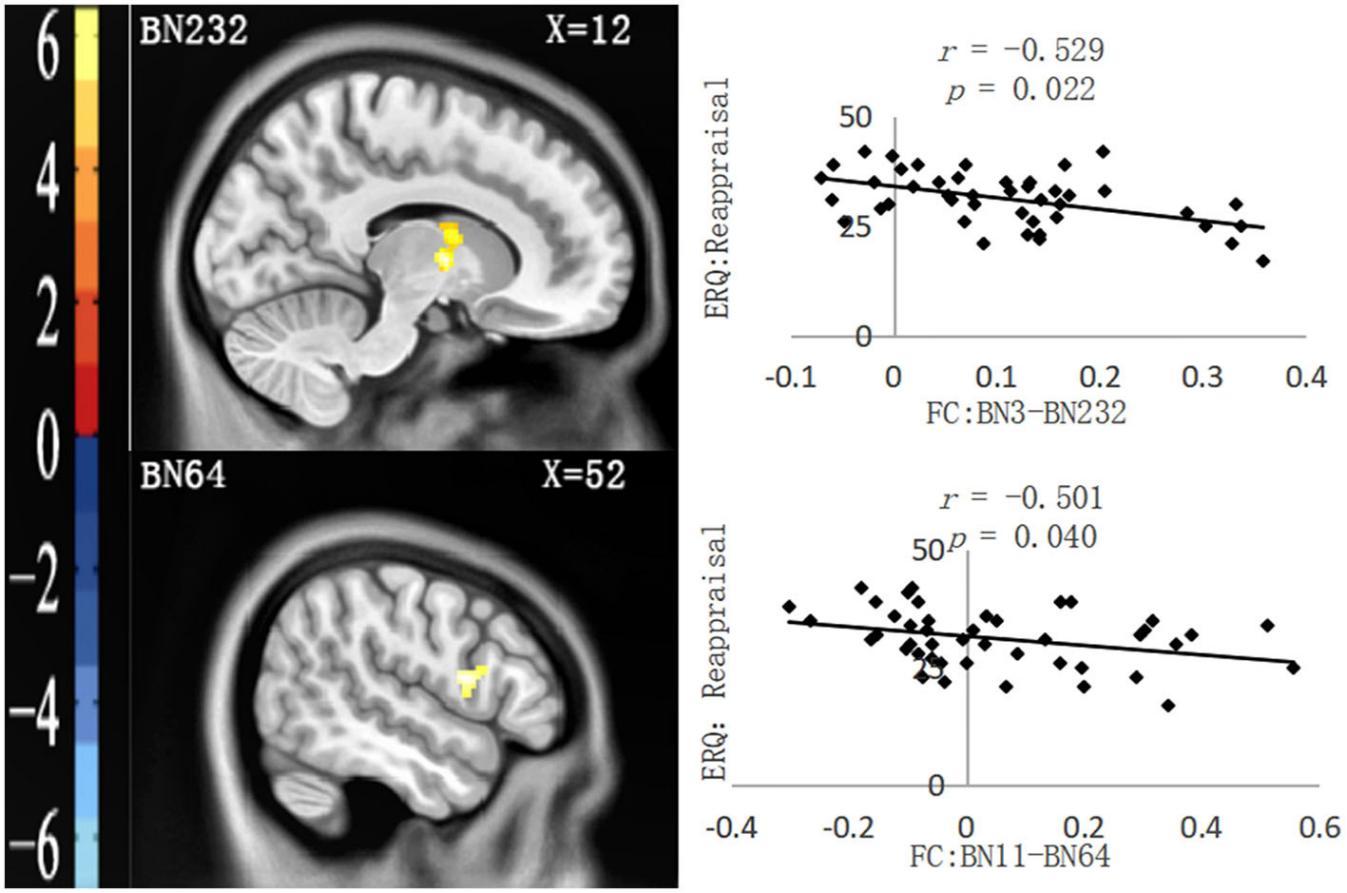
Functional connections (neglect group > control group) significantly correlated with ERQ: reappraisal. **Left**: Brain regions functionally connected with the ROIs. **Right**: Scatter plot of the correlation between the FC value and ERQ reappraisal. All the *p-*values underwent Bonferroni corrections.

**Figure 2:**
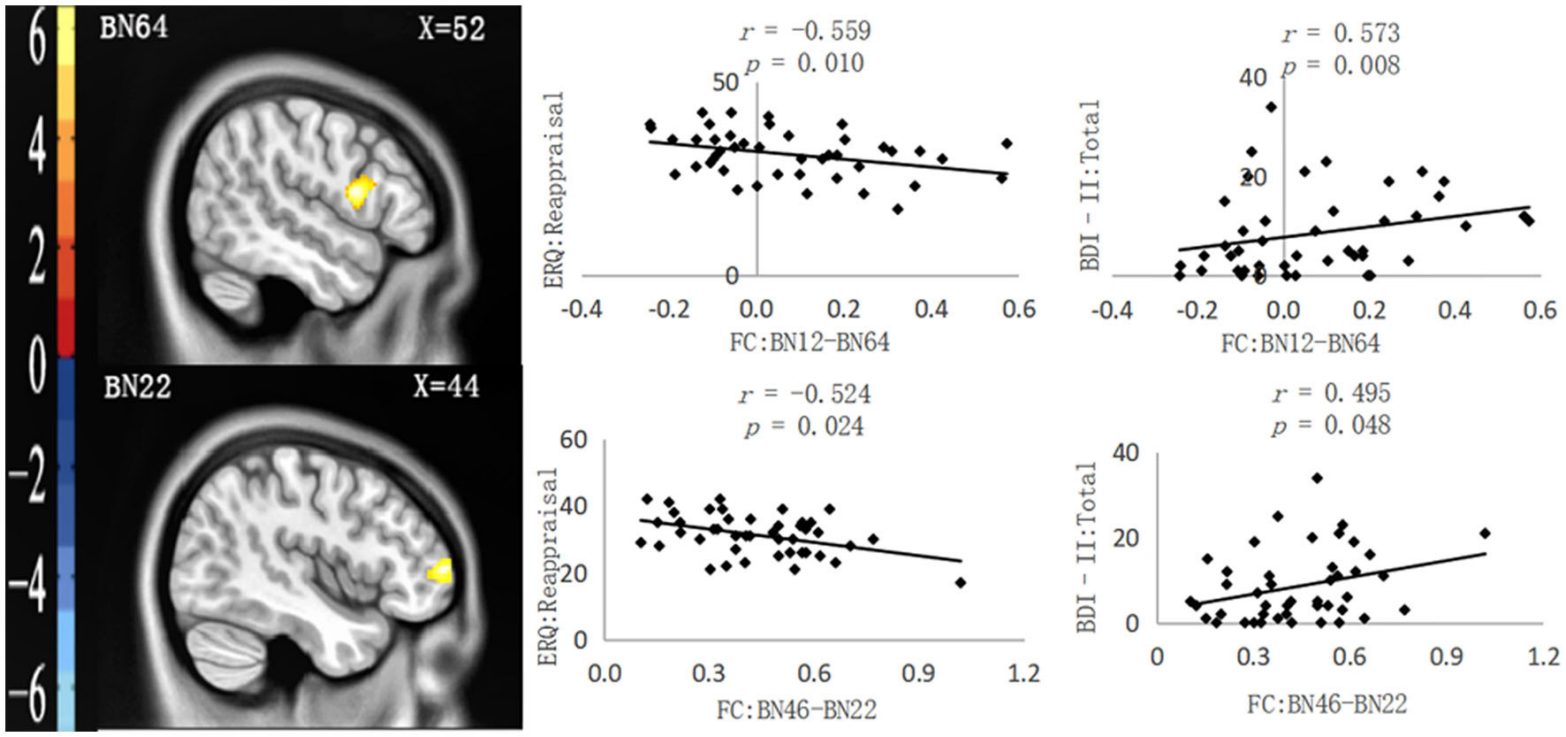
Functional connections (neglect group > control group) significantly correlated with ERQ: Reappraisal and BDI-II: Total. **Left**: Brain regions functionally connected with the ROIs. **Right**: Scatter plot of the correlation between the FC value and ERQ reappraisal and BDI-II: Total. All the *p-*values underwent Bonferroni corrections.

As shown in Table 2, four functional connection values between PFC subregions and other brain regions significantly correlated with questionnaire data. After controlling for all covariates, partial correlation analysis showed that two functional connection values were only significantly negatively correlated with the ERQ reappraisal score, namely, FC: BN3–BN232 (the right thalamus in the AAL Atlas) and FC: BN11–BN64 (the right Rolandic oper in the AAL Atlas), as shown in Figure 1. Moreover, two functional connection values were significantly negatively correlated with the ERQ reappraisal score and significantly positively correlated with the BDI-II total score, namely, FC: BN12–BN64 (right Rolandic Oper in AAL Atlas) and FC: BN46–BN22 (right Frontal Mid in AAL Atlas), as shown in Figure 2. Functional connection values were not significantly correlated with the ERQ suppression score.

**Table 2:** Functional connections (neglect group > control group) significantly correlated with the questionnaire data (*n* = 45).

**ROI**	**Brain regions functionally connected with the ROI**		**Voxels**	***t***	**MNI coordinates (x, y, z)**
	**Label ID**	**Gyrus**			
BN3 (Left superior frontal gyrus)	232	Right thalamus	208	5.92	12, −2, 4
BN11 (Left superior frontal gyrus)	64	Right precentral gyrus	126	4.61	52, 8, 14
BN12 (Right superior frontal gyrus)	64	Right precentral gyrus	232	6.30	52, 8, 14
BN46 (Right orbital gyrus)	22	Right middle frontal gyrus	236	5.69	44, 56, 6

According to the results of partial correlation analysis, taking the ERQ reappraisal score and the BDI-II total score as dependent variables, the functional connection values significantly correlated with the two scores and all the covariates as common independent variables, and a linear regression analysis was conducted. It was found that only FC: BN46–BN22 was significantly negatively correlated with the ERQ reappraisal score [Beta = −0.603, *t* = −3.312, *p* = 0.004, 95.0% confidence interval (−31.037, −7.001)] and significantly positively correlated with the BDI-II total score [Beta = 0.477, *t* = 2.750, *p* = 0.012, 95.0% confidence interval (5.013, 36.131)].

**Figure 3:**
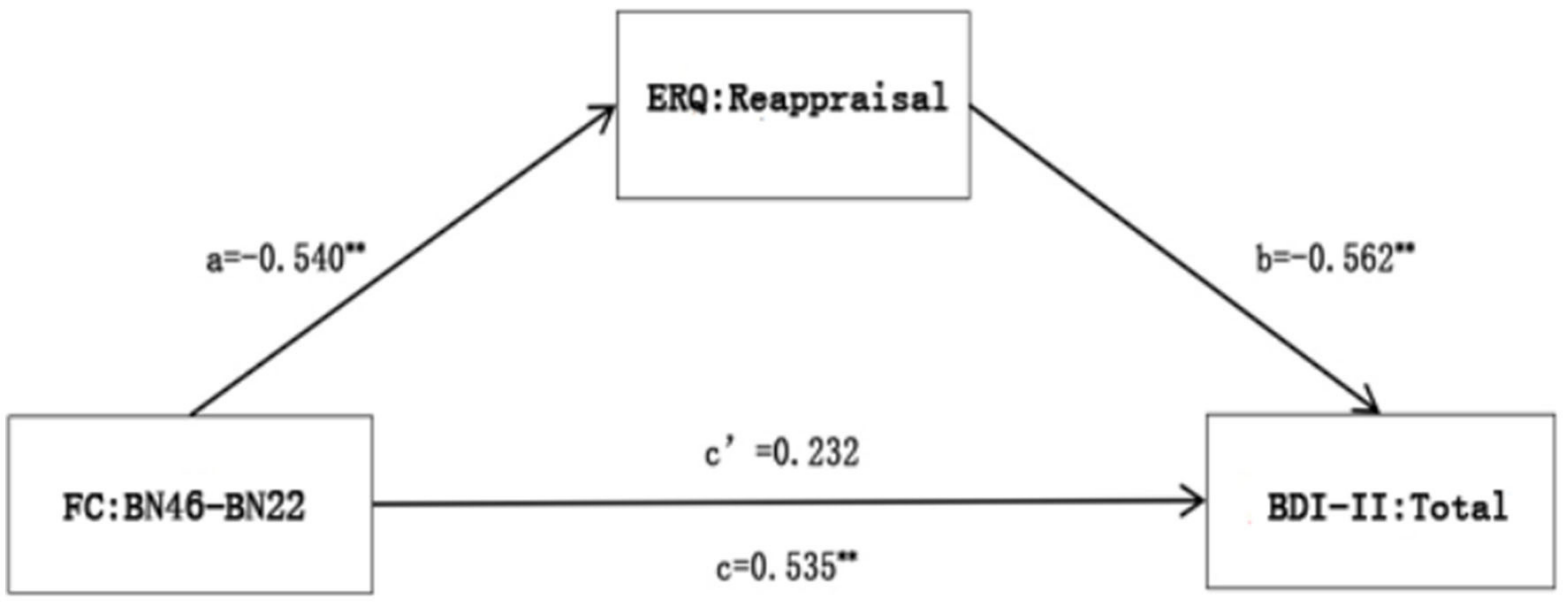
A mediation analysis of FC: BN46-BN22 with ERQ reappraisal and BDI-II total. **p < 0.01.

The results of linear regression analysis indicated that by taking FC: BN46–BN22 as the independent variable, the BDI-II total score as the dependent variable, and the ERQ reappraisal score as the mediating variable, a mediation analysis was conducted, controlling all covariates. The indirect and total effects were significant, suggesting that the ERQ reappraisal score wholly mediated the relationship between FC: BN46–BN22 and the BDI-II total score, as seen in Figure 3.

Logically speaking, the functional connections of PFC may also mediate the relationship between the ERQ reappraisal score and the BDI-II total score, so the following steps were attempted. By taking the ERQ reappraisal score as the independent variable, the BDI-II total score as the dependent variable, and FC: BN46–BN22 as the mediating variable, a mediation analysis was conducted, controlling all covariates. The results showed that FC: BN46–BN22 could not mediate the relationship between the ERQ reappraisal score and the BDI-II total score, as shown in Appendix 5.

## 5. Discussion

This study investigated the relationships between the tendency to utilize emotion regulation strategies, depressive symptoms, and the functional connections of the PFC in college students with primary CEN, based on a questionnaire survey and the rs-fMRI technique. The questionnaire survey results showed that the BDI-II total score in the neglect group was significantly higher than that in the control group, and the ERQ reappraisal score in the neglect group was considerably lower than that in the control group. However, the two groups had no significant difference in the ERQ suppression score. Brain-behavior correlation analysis revealed that the functional connection value of BN46 with BN22 was significantly negatively correlated with the ERQ reappraisal score and significantly positively correlated with the BDI-II total score; the ERQ reappraisal score was significantly negatively correlated with the BDI-II total score. The ERQ reappraisal score wholly mediated the relationship between the functional connection of BN46 with BN22 and the BDI-II total score.

### 5.1. Relationships among primary CEN, emotion regulation, and depressive symptoms

Shipman et al. (2005) found that children who experienced chronic emotional neglect tended to develop psychological disorders in adulthood due to the less utilization of adaptive emotion regulation skills such as reappraisal. Wang et al. (2017) also found that adults with depression had experienced CEN, and this experience was correlated with the tendency to utilize adaptive emotion regulation strategies less frequently. These results provide a reliable basis for the view that relationships may exist between CEN, the tendency to utilize emotion regulation strategies, and depressive symptoms in adulthood. In the present study, taking healthy college students with primary CEN, the BDI-II total score in the neglect group was significantly higher than that in the control group, and the ERQ reappraisal score was considerably lower than that in the control group. Moreover, the ERQ reappraisal score was significantly negatively correlated with the BDI-II total score, further verifying the above views.

According to Gross's emotion regulation process model, reappraisal may be more adaptive and protective than suppression. Reappraisal is one of the antecedent-focused emotion regulation strategies because it focuses on helping individuals reinterpret and understand the nature and significance of the situation or stimulus that triggers their emotions and makes them take action at the early stage of the emotion generation process (Gross, 2002). The aforementioned characteristics of reappraisal make it possible to effectively control improper emotional expression, improve the bad subjective emotional experience, and simultaneously reduce an excessive emotional physiological response (Gross, 1998; Ochsner et al., 2002; Ray et al., 2010; Kim and Hamann, 2012). Individuals who often use the reappraisal strategy have better emotional and mental health (Gross and John, 2003). Compared with reappraisal, suppression may only forcibly reduce the impulse of negative emotional expression. For individuals who often use the suppression strategy, their negative experience and corresponding physiological response may not be effectively improved (Gross and Levenson, 1997; Harris, 2001).

The results suggest that although primary CEN may not necessarily induce depression that meets the clinical diagnostic criteria, it may be more prone to related depression symptoms. Because these individuals may choose the reappraisal strategy less frequently to deal with significant emotional events, it is also necessary for college educators to pay attention to the daily emotional state of these individuals to avoid the occurrence of psychological crises.

### 5.2. The mediating role of the reappraisal strategy in the relationship between the functional connections of PFC and depressive symptoms

Previous studies found that depression patients with CEN had abnormal brain functional connections related to emotion regulation, particularly the PFC (Wang et al., 2014; Souza-Queiroz et al., 2016; Duque-Alarcón et al., 2019). Based on the result from questionnaire data in this study, we further explored the relationships among brain functional connections in the resting state, emotion regulation strategies, and the depressive symptoms of individuals with primary CEN. The results showed that compared with the control group, the neglect group had a more robust functional connection between BN46 (right orbital gyrus) and BN22 (right middle frontal gyrus). The value of the functional connection was significantly negatively correlated with the ERQ reappraisal score and significantly positively correlated with the BDI-II total score. Furthermore, mediation analysis further found that the ERQ reappraisal score wholly mediated the relationship between the functional connection and the BDI-II total score. These findings are the first to fully reveal the relationships among CEN, emotion regulation strategies, depressive symptoms, and resting-state brain function in adulthood.

It has been suggested that the orbitofrontal cortex (OFC) plays an essential role in receiving and processing emotional information and expressing and controlling dynamic behavior by suppressing unwanted or uncomfortable feelings and related neural activities (Shimamura, 2000). However, some studies revealed that OFC had a top-down suppressing effect on the amygdala affecting negative emotional symptoms (Price, 2007; Salzman and Fusi, 2010). For example, Zhang et al. (2014) found that the functional connections of OFC with the amygdala in patients with MDD were significantly decreased compared with those of the control group. There is growing evidence that the middle frontal gyrus (MFG) could regulate the function of OFC. MFG is a part of the dorsal attention network (DAN), which participates in goal-oriented top-down processing and plays a crucial role in emotion regulation tasks involving high-order cognition (such as utilizing the reappraisal strategy) (Corbetta and Shulman, 2002). The changes in the activity in MGF may affect the evaluation and feedback process of the emotional stimulus of OFC, leading to possible emotional disorders (Ochsner et al., 2004; Eippert et al., 2007). For example, Xu et al. (2017) found that compared with healthy people, people with schizophrenia exhibited a series of cognitive and emotional disorders, and the functional connections of the bilateral orbitofrontal gyrus with right MGF significantly decreased.

Interestingly, this study found the prefrontal functional connection to be significantly positively correlated with the score of depressive symptoms, which was inconsistent with some previous study results. For example, Wang et al. (2014) found that the prefrontal functional connections of patients with MDD significantly got reduced compared with that of healthy people. One possible explanation for these differences is that the subjects in those studies were patients diagnosed with depression or other psychological diseases. In addition, even among patients with depression, the prefrontal lobe activity or the strength of functional connections may not be reduced. Xu et al. (2019) found that compared with healthy people, the ALLF of the left inferior frontal gyrus orbital in patients with MDD significantly increased. It is speculated that the neglect group in this study had more robust prefrontal functional connections and more depressive symptoms, which may be a compensatory mechanism developed by them to offset the increase of depressive symptoms related to CEN.

### 5.3. Limitations

There are some limitations in this study. (1) It is difficult to obtain the dynamic changes of the process of the emotion regulation ability, depressive symptoms, and neural activities during the period of experiencing CEN and later the longer life cycle, especially the relationship between the time factor and the above variables. (2) To manipulate the variable of CEN better, the inclusion criteria of the control group adopted the strict standard of no childhood traumatic experience, but this also blocked the possibility of taking CEN as a continuous variable to investigate its relationship with emotion regulation strategies and depressive symptoms. (3) The data analysis method of rs-fMRI is not comprehensive. For example, the relationships among brain local functional activities (ALFF and ReHo), the topological attributes of the brain network and emotion regulation strategies, and depressive symptoms have not been investigated. (4) The multiple correction standard used for brain imaging analysis was not stringent enough. In the future, it is necessary to expand the age range of subjects, make more effective screening by the extensive use of multiple tools, and use more comprehensive data analysis methods to explore the relationships among primary CEN and emotion regulation, depressive symptoms, as well as the functional brain activity.

## 6. Conclusion

For the first time, this study investigated the relationships among primary CEN, adulthood emotion regulation strategies, depression symptoms, and prefrontal functional connections. The results showed that college students with primary CEN utilized the reappraisal less frequently and had more depressive symptoms and more robust prefrontal functional connections. Moreover, the tendency to utilize the reappraisal strategy mediated the relationship between prefrontal functional connections and depression symptoms. The results suggest that primary CEN may closely correlate with more depressive symptoms in adulthood. Moreover, the more robust spontaneous activity of the prefrontal lobe may also be closely associated with more depressive symptoms by utilizing the reappraisal strategy less frequently.

## Data availability statement

The raw data supporting the conclusions of this article will be made available by the authors, without undue reservation.

## Ethics statement

The studies involving human participants were reviewed and approved by the Ethics Committee of Tianjin Normal University. The patients/participants provided their written informed consent to participate in this study. Written informed consent was obtained from the individual(s) for the publication of any potentially identifiable images or data included in this article.

## Author contributions

All authors were involved in the statistical analyses and data collection of the study. All authors contributed and have approved the final manuscript.
