# Supplementary material for: The relationship between childhood emotional neglect experience and depressive symptoms and prefrontal resting functional connections in college students: The mediating role of reappraisal strategy
Source: Front Behav Neurosci. 2023 Feb 22;17:927389. doi: 10.3389/fnbeh.2023.927389 (PMC10037214; doi:10.3389/fnbeh.2023.927389)
Supplement: Supplementary file 1 [file Data_Sheet_1.docx]

**Appendix**

**Appendix 1. Numbers of participants retained and removed According to head movement**

All the numbers of participants were 001-053, among which 001, 009, 019, 024, 033 and 042 fell off in advance and did not participate in the formal experiment.

The Numbers retained in the neglect group: 002，003，005，006，007，010，012，013，014，015，017，018，025，026，032，034，037，048，050；

The Numbers removed from the neglect group: 016，046;

The Numbers retained in the control group: 004，011，020，021，022，023，027，028，029，030，031，035，036，038，039，040，041，043，044，045，047，049，051，052，053；

The Numbers removed from the control group: 008

**Appendix 2. BN Atlas subregions**

| **Lobe** | **Gyrus** | **Left and Right**  **Hemisphere** | **Label ID.L** | **Label ID.R** | **Anatomical and modified Cyto-architectonic descriptions** | **lh.MNI**  **(X,Y,Z)** | **rh.MNI**  **(X,Y,Z)** |
| --- | --- | --- | --- | --- | --- | --- | --- |
| **Frontal Lobe** | SFG, Superior Frontal Gyrus | SFG_L(R)_7_1 | 1 | 2 | A8m, medial area 8 | -5 ,15, 54 | 7, 16, 54 |
|  |  | SFG_L(R)_7_2 | 3 | 4 | A8dl, dorsolateral area 8 | -18, 24, 53 | 22, 26, 51 |
|  |  | SFG_L(R)_7_3 | 5 | 6 | A9l, lateral area 9 | -11, 49, 40 | 13, 48, 40 |
|  |  | SFG_L(R)_7_4 | 7 | 8 | A6dl, dorsolateral area 6 | -18, -1, 65 | 20, 4, 64 |
|  |  | SFG_L(R)_7_5 | 9 | 10 | A6m, medial area 6 | -6, -5, 58 | 7, -4, 60 |
|  |  | SFG_L(R)_7_6 | 11 | 12 | A9m,medial area 9 | -5, 36, 38 | 6, 38, 35 |
|  |  | SFG_L(R)_7_7 | 13 | 14 | A10m, medial area 10 | -8, 56, 15 | 8, 58, 13 |
|  | MFG,  Middle Frontal Gyrus | MFG_L(R)_7_1 | 15 | 16 | A9/46d, dorsal area 9/46 | -27, 43, 31 | 30, 37, 36 |
|  |  | MFG_L(R)_7_2 | 17 | 18 | IFJ, inferior frontal junction | -42, 13, 36 | 42, 11, 39 |
|  |  | MFG_L(R)_7_3 | 19 | 20 | A46, area 46 | -28, 56, 12 | 28, 55, 17 |
|  |  | MFG_L(R)_7_4 | 21 | 22 | A9/46v, ventral area 9/46 | -41, 41, 16 | 42, 44, 14 |
|  |  | MFG_L(R)_7_5 | 23 | 24 | A8vl, ventrolateral area 8 | -33, 23, 45 | 42, 27, 39 |
|  |  | MFG_L(R)_7_6 | 25 | 26 | A6vl, ventrolateral area 6 | -32, 4, 55 | 34, 8, 54 |
|  |  | MFG_L(R)_7_7 | 27 | 28 | A10l, lateral area10 | -26, 60, -6 | 25, 61, -4 |
|  | IFG, Inferior Frontal Gyrus | IFG_L(R)_6_1 | 29 | 30 | A44d,dorsal area 44 | -46, 13, 24 | 45, 16, 25 |
|  |  | IFG_L(R)_6_2 | 31 | 32 | IFS, inferior frontal sulcus | -47, 32, 14 | 48, 35, 13 |
|  |  | IFG_L(R)_6_3 | 33 | 34 | A45c, caudal area 45 | -53, 23, 11 | 54, 24, 12 |
|  |  | IFG_L(R)_6_4 | 35 | 36 | A45r, rostral area 45 | -49, 36, -3 | 51, 36, -1 |
|  |  | IFG_L(R)_6_5 | 37 | 38 | A44op, opercular area 44 | -39, 23, 4 | 42, 22, 3 |
|  |  | IFG_L(R)_6_6 | 39 | 40 | A44v, ventral area 44 | -52, 13, 6 | 54, 14, 11 |
|  | OrG, Orbital Gyrus | OrG_L(R)_6_1 | 41 | 42 | A14m, medial area 14 | -7, 54, -7 | 6, 47, -7 |
|  |  | OrG_L(R)_6_2 | 43 | 44 | A12/47o, orbital area 12/47 | -36, 33, -16 | 40, 39, -14 |
|  |  | OrG_L(R)_6_3 | 45 | 46 | A11l, lateral area 11 | -23, 38, -18 | 23, 36, -18 |
|  |  | OrG_L(R)_6_4 | 47 | 48 | A11m, medial area 11 | -6, 52, -19 | 6, 57, -16 |
|  |  | OrG_L(R)_6_5 | 49 | 50 | A13, area 13 | -10, 18, -19 | 9, 20, -19 |
|  |  | OrG_L(R)_6_6 | 51 | 52 | A12/47l, lateral area 12/47 | -41, 32, -9 | 42, 31, -9 |
|  | PrG, Precentral Gyrus | PrG_L(R)_6_1 | 53 | 54 | A4hf, area 4(head and face region) | -49, -8, 39 | 55, -2, 33 |
|  |  | PrG_L(R)_6_2 | 55 | 56 | A6cdl, caudal dorsolateral area 6 | -32, -9, 58 | 33, -7, 57 |
|  |  | PrG_L(R)_6_3 | 57 | 58 | A4ul, area 4(upper limb region) | -26, -25, 63 | 34, -19, 59 |
|  |  | PrG_L(R)_6_4 | 59 | 60 | A4t, area 4(trunk region) | -13, -20, 73 | 15, -22, 71 |
|  |  | PrG_L(R)_6_5 | 61 | 62 | A4tl, area 4  (tongue and larynx region) | -52, 0, 8 | 54, 4, 9 |
|  |  | PrG_L(R)_6_6 | 63 | 64 | A6cvl, caudal ventrolateral area 6 | -49, 5, 30 | 51, 7, 30 |
|  | PCL,  Paracentral Lobule | PCL_L(R)_2_1 | 65 | 66 | A1/2/3ll, area1/2/3  (lower limb region) | -8, -38, 58 | 10, -34, 54 |
|  |  | PCL_L(R)_2_2 | 67 | 68 | A4ll, area 4, (lower limb region) | -4, -23, 61 | 5, -21, 61 |
| **Temporal Lobe** | STG, Superior Temporal Gyrus | STG_L(R)_6_1 | 69 | 70 | A38m, medial area 38 | -32, 14, -34 | 31, 15, -34 |
|  |  | STG_L(R)_6_2 | 71 | 72 | A41/42, area 41/42 | -54, -32, 12 | 54, -24, 11 |
|  |  | STG_L(R)_6_3 | 73 | 74 | TE1.0 and TE1.2 | -50, -11, 1 | 51, -4, -1 |
|  |  | STG_L(R)_6_4 | 75 | 76 | A22c, caudal area 22 | -62, -33, 7 | 66, -20, 6 |
|  |  | STG_L(R)_6_5 | 77 | 78 | A38l, lateral area 38 | -45, 11, -20 | 47, 12, -20 |
|  |  | STG_L(R)_6_6 | 79 | 80 | A22r, rostral area 22 | -55, -3, -10 | 56, -12, -5 |
|  | MTG, Middle Temporal Gyrus | MTG_L(R)_4_1 | 81 | 82 | A21c, caudal area 21 | -65, -30, -12 | 65, -29, -13 |
|  |  | MTG_L(R)_4_2 | 83 | 84 | A21r, rostral area 21 | -53, 2, -30 | 51, 6, -32 |
|  |  | MTG_L(R)_4_3 | 85 | 86 | A37dl, dorsolateral area37 | -59, -58, 4 | 60, -53, 3 |
|  |  | MTG_L(R)_4_4 | 87 | 88 | aSTS,  anterior superior temporal sulcus | -58, -20, -9 | 58, -16, -10 |
|  | ITG, Inferior Temporal Gyrus | ITG_L(R)_7_1 | 89 | 90 | A20iv, intermediate ventral area 20 | -45, -26, -27 | 46, -14, -33 |
|  |  | ITG_L(R)_7_2 | 91 | 92 | A37elv, extreme lateroventral area37 | -51, -57, -15 | 53, -52, -18 |
|  |  | ITG_L(R)_7_3 | 93 | 94 | A20r, rostral area 20 | -43, -2, -41 | 40, 0, -43 |
|  |  | ITG_L(R)_7_4 | 95 | 96 | A20il, intermediate lateral area 20 | -56, -16, -28 | 55, -11, -32 |
|  |  | ITG_L(R)_7_5 | 97 | 98 | A37vl, ventrolateral area 37 | -55, -60, -6 | 54, -57, -8 |
|  |  | ITG_L(R)_7_6 | 99 | 100 | A20cl, caudolateral of area 20 | -59, -42, -16 | 61, -40, -17 |
|  |  | ITG_L(R)_7_7 | 101 | 102 | A20cv, caudoventral of area 20 | -55, -31, -27 | 54, -31, -26 |
|  | FuG, Fusiform Gyrus | FuG_L(R)_3_1 | 103 | 104 | A20rv, rostroventral area 20 | -33, -16, -32 | 33, -15, -34 |
|  |  | FuG_L(R)_3_2 | 105 | 106 | A37mv, medioventral area37 | -31, -64, -14 | 31, -62, -14 |
|  |  | FuG_L(R)_3_3 | 107 | 108 | A37lv, lateroventral area37 | -42, -51, -17 | 43, -49, -19 |
|  | PhG,  Parahippocampal Gyrus | PhG_L(R)_6_1 | 109 | 110 | A35/36r, rostral area 35/36 | -27, -7, -34 | 28, -8, -33 |
|  |  | PhG_L(R)_6_2 | 111 | 112 | A35/36c, caudal area 35/36 | -25, -25, -26 | 26, -23, -27 |
|  |  | PhG_L(R)_6_3 | 113 | 114 | TL, area TL (lateral PPHC, posterior parahippocampal gyrus) | -28, -32, -18 | 30, -30, -18 |
|  |  | PhG_L(R)_6_4 | 115 | 116 | A28/34, area 28/34  (EC, entorhinal cortex) | -19, -12, -30 | 19, -10, -30 |
|  |  | PhG_L(R)_6_5 | 117 | 118 | TI, area TI(temporal agranular insular cortex) | -23, 2, -32 | 22, 1, -36 |
|  |  | PhG_L(R)_6_6 | 119 | 120 | TH, area TH (medial PPHC) | -17, -39, -10 | 19, -36, -11 |
|  | pSTS, posterior Superior Temporal Sulcus | pSTS_L(R)_2_1 | 121 | 122 | rpSTS, rostroposterior superior temporal sulcus | -54, -40, 4 | 53, -37, 3 |
|  |  | pSTS_L(R)_2_2 | 123 | 124 | cpSTS, caudoposterior superior temporal sulcus | -52, -50, 11 | 57, -40, 12 |
| **Parietal Lobe** | SPL, Superior Parietal Lobule | SPL_L(R)_5_1 | 125 | 126 | A7r, rostral area 7 | -16, -60, 63 | 19, -57, 65 |
|  |  | SPL_L(R)_5_2 | 127 | 128 | A7c, caudal area 7 | -15, -71, 52 | 19, -69, 54 |
|  |  | SPL_L(R)_5_3 | 129 | 130 | A5l, lateral area 5 | -33, -47, 50 | 35, -42, 54 |
|  |  | SPL_L(R)_5_4 | 131 | 132 | A7pc, postcentral area 7 | -22, -47, 65 | 23, -43, 67 |
|  |  | SPL_L(R)_5_5 | 133 | 134 | A7ip, intraparietal area 7(hIP3) | -27, -59, 54 | 31, -54, 53 |
|  | IPL, Inferior Parietal Lobule | IPL_L(R)_6_1 | 135 | 136 | A39c, caudal area 39(PGp) | -34, -80, 29 | 45, -71, 20 |
|  |  | IPL_L(R)_6_2 | 137 | 138 | A39rd, rostrodorsal area 39(Hip3) | -38, -61, 46 | 39, -65, 44 |
|  |  | IPL_L(R)_6_3 | 139 | 140 | A40rd, rostrodorsal area 40(PFt) | -51, -33, 42 | 47, -35, 45 |
|  |  | IPL_L(R)_6_4 | 141 | 142 | A40c, caudal area 40(PFm) | -56, -49, 38 | 57, -44, 38 |
|  |  | IPL_L(R)_6_5 | 143 | 144 | A39rv, rostroventral area 39(PGa) | -47, -65, 26 | 53, -54, 25 |
|  |  | IPL_L(R)_6_6 | 145 | 146 | A40rv, rostroventral area 40(PFop) | -53, -31, 23 | 55, -26, 26 |
|  | Pcun, Precuneus | PCun_L(R)_4_1 | 147 | 148 | A7m, medial area 7(PEp) | -5, -63, 51 | 6, -65, 51 |
|  |  | PCun_L(R)_4_2 | 149 | 150 | A5m, medial area 5(PEm) | -8, -47, 57 | 7, -47, 58 |
|  |  | PCun_L(R)_4_3 | 151 | 152 | dmPOS, dorsomedial parietooccipital sulcus(PEr) | -12, -67, 25 | 16, -64, 25 |
|  |  | PCun_L(R)_4_4 | 153 | 154 | A31, area 31 (Lc1) | -6, -55, 34 | 6, -54, 35 |
|  | PoG  Postcentral Gyrus | PoG_L(R)_4_1 | 155 | 156 | A1/2/3ulhf, area 1/2/3  (upper limb, head and face region) | -50, -16, 43 | 50, -14, 44 |
|  |  | PoG_L(R)_4_2 | 157 | 158 | A1/2/3tonIa, area 1/2/3(tongue and larynx region) | -56, -14, 16 | 56, -10, 15 |
|  |  | PoG_L(R)_4_3 | 159 | 160 | A2, area 2 | -46, -30, 50 | 48, -24, 48 |
|  |  | PoG_L(R)_4_4 | 161 | 162 | A1/2/3tru, area1/2/3(trunk region) | -21, -35, 68 | 20, -33, 69 |
| **InsularLobe** | INS, Insular Gyrus | INS_L(R)_6_1 | 163 | 164 | G, hypergranular insula | -36, -20, 10 | 37, -18, 8 |
|  |  | INS_L(R)_6_2 | 165 | 166 | vIa, ventral agranular insula | -32, 14, -13 | 33, 14, -13 |
|  |  | INS_L(R)_6_3 | 167 | 168 | dIa, dorsal agranular insula | -34, 18, 1 | 36, 18, 1 |
|  |  | INS_L(R)_6_4 | 169 | 170 | vId/vIg, ventral dysgranular and granular insula | -38, -4, -9 | 39, -2, -9 |
|  |  | INS_L(R)_6_5 | 171 | 172 | dIg, dorsal granular insula | -38, -8, 8 | 39, -7, 8 |
|  |  | INS_L(R)_6_6 | 173 | 174 | dId, dorsal dysgranular insula | -38, 5, 5 | 38, 5, 5 |
| **Limbic Lobe** | CG, Cingulate Gyrus | CG_L(R)_7_1 | 175 | 176 | A23d, dorsal area 23 | -4, -39, 31 | 4, -37, 32 |
|  |  | CG_L(R)_7_2 | 177 | 178 | A24rv, rostroventral area 24 | -3, 8, 25 | 5, 22, 12 |
|  |  | CG_L(R)_7_3 | 179 | 180 | A32p, pregenual area 32 | -6, 34, 21 | 5, 28, 27 |
|  |  | CG_L(R)_7_4 | 181 | 182 | A23v, ventral area 23 | -8, -47, 10 | 9, -44, 11 |
|  |  | CG_L(R)_7_5 | 183 | 184 | A24cd, caudodorsal area 24 | -5, 7, 37 | 4, 6, 38 |
|  |  | CG_L(R)_7_6 | 185 | 186 | A23c, caudal area 23 | -7, -23, 41 | 6, -20, 40 |
|  |  | CG_L(R)_7_7 | 187 | 188 | A32sg, subgenual area 32 | -4, 39, -2 | 5, 41, 6 |
| **Occipital Lobe** | MVOcC  MedioVentral Occipital Cortex | MVOcC _L(R)_5_1 | 189 | 190 | cLinG, caudal lingual gyrus | -11, -82, -11 | 10, -85, -9 |
|  |  | MVOcC _L(R)_5_2 | 191 | 192 | rCunG, rostral cuneus gyrus | -5, -81, 10 | 7, -76, 11 |
|  |  | MVOcC _L(R)_5_3 | 193 | 194 | cCunG, caudal cuneus gyrus | -6, -94, 1 | 8, -90, 12 |
|  |  | MVOcC _L(R)_5_4 | 195 | 196 | rLinG, rostral lingual gyrus | -17, -60, -6 | 18, -60, -7 |
|  |  | MVOcC _L(R)_5_5 | 197 | 198 | vmPOS,ventromedial parietooccipital sulcus | -13, -68, 12 | 15, -63, 12 |
|  | LOcC, lateral Occipital Cortex | LOcC_L(R)_4_1 | 199 | 200 | mOccG, middle occipital gyrus | -31, -89, 11 | 34, -86, 11 |
|  |  | LOcC _L(R)_4_2 | 201 | 202 | V5/MT+, area V5/MT+ | -46, -74, 3 | 48, -70, -1 |
|  |  | LOcC _L(R)_4_3 | 203 | 204 | OPC, occipital polar cortex | -18, -99, 2 | 22, -97, 4 |
|  |  | LOcC_L(R)_4_4 | 205 | 206 | iOccG, inferior occipital gyrus | -30, -88, -12 | 32, -85, -12 |
|  |  | LOcC _L(R)_2_1 | 207 | 208 | msOccG, medial superior occipital gyrus | -11, -88, 31 | 16, -85, 34 |
|  |  | LOcC _L(R)_2_2 | 209 | 210 | lsOccG, lateral superior occipital gyrus | -22, -77, 36 | 29, -75, 36 |
| **Subcortical Nuclei** | Amyg, Amygdala | Amyg_L(R)_2_1 | 211 | 212 | mAmyg, medial amygdala | -19, -2, -20 | 19, -2, -19 |
|  |  | Amyg_L(R)_2_2 | 213 | 214 | lAmyg, lateral amygdala | -27, -4, -20 | 28, -3, -20 |
|  | Hipp,  Hippocampus | Hipp_L(R)_2_1 | 215 | 216 | rHipp, rostral hippocampus | -22, -14, -19 | 22, -12, -20 |
|  |  | Hipp_L(R)_2_2 | 217 | 218 | cHipp, caudal hippocampus | -28, -30, -10 | 29, -27, -10 |
|  | BG,  Basal  Ganglia | BG_L(R)_6_1 | 219 | 220 | vCa, ventral caudate | -12, 14, 0 | 15, 14, -2 |
|  |  | BG_L(R)_6_2 | 221 | 222 | GP, globus pallidus | -22, -2, 4 | 22, -2, 3 |
|  |  | BG_L(R)_6_3 | 223 | 224 | NAC, nucleus accumbens | -17, 3, -9 | 15, 8, -9 |
|  |  | BG_L(R)_6_4 | 225 | 226 | vmPu, ventromedial putamen | -23, 7, -4 | 22, 8, -1 |
|  |  | BG_L(R)_6_5 | 227 | 228 | dCa, dorsal caudate | -14, 2, 16 | 14, 5, 14 |
|  |  | BG_L(R)_6_6 | 229 | 230 | dlPu, dorsolateral putamen | -28, -5, 2 | 29, -3, 1 |
|  | Tha, Thalamus | Tha_L(R)_8_1 | 231 | 232 | mPFtha, medial pre-frontal thalamus | -7, -12, 5 | 7, -11, 6 |
|  |  | Tha_L(R)_8_2 | 233 | 234 | mPMtha, pre-motor thalamus | -18, -13, 3 | 12, -14, 1 |
|  |  | Tha_L(R)_8_3 | 235 | 236 | Stha, sensory thalamus | -18, -23, 4 | 18, -22, 3 |
|  |  | Tha_L(R)_8_4 | 237 | 238 | rTtha, rostral temporal thalamus | -7, -14, 7 | 3, -13, 5 |
|  |  | Tha_L(R)_8_5 | 239 | 240 | PPtha, posterior parietal thalamus | -16, -24, 6 | 15, -25, 6 |
|  |  | Tha_L(R)_8_6 | 241 | 242 | Otha, occipital thalamus | -15, -28, 4 | 13, -27, 8 |
|  |  | Tha_L(R)_8_7 | 243 | 244 | cTtha, caudal temporal thalamus | -12, -22, 13 | 10, -14, 14 |
|  |  | Tha_L(R)_8_8 | 245 | 246 | lPFtha, lateral pre-frontal thalamus | -11, -14, 2 | 13, -16, 7 |

**Appendix 3. Differences in demographic data between the two groups**

| Variables |  | Neglect Group (*n*=21) | Control Group (*n*=26) | *t* / *χ^2^* | *p* |
| --- | --- | --- | --- | --- | --- |
| Age |  | 19.19±0.68 | 19.04±0.87 | *t*=0.66 | *p*=0.52 |
| Gender |  |  |  | *χ^2^*=0.026 | *p*=0.872 |
|  | Male | 10 | 13 |  |  |
|  | Female | 11 | 13 |  |  |
| Education Levels of Parents |  |  |  | *χ^2^*=3.594/2.280 | *p*=0.309/0.516 |
|  | None | 0/0 | 0/0 |  |  |
|  | Primary School | 1/6 | 1/3 |  |  |
|  | Junior Middle School | 12/6 | 8/10 |  |  |
|  | High School/  Technical Secondary School | 6/6 | 12/8 |  |  |
|  | University / Junior College | 2/3 | 5/5 |  |  |
|  | Master Degree or Above | 0/0 | 0/0 |  |  |
| Work Status of Parents |  |  |  | *χ^2^*=3.460/1.918 | *p*=0.749/0.927 |
|  | None | 2/5 | 1/5 |  |  |
|  | Education System | 0/1 | 1/1 |  |  |
|  | Health System | 0/1 | 1/1 |  |  |
|  | State Owned Enterprises | 2/1 | 5/1 |  |  |
|  | Government Departments | 1/3 | 2/2 |  |  |
|  | Private Enterprises | 3/1 | 3/4 |  |  |
|  | Others | 13/9 | 13/12 |  |  |
| Family Economic Status |  |  |  | *χ^2^*=10.692 | *p*=0.005^**^ |
|  | poor | 7 | 0 |  |  |
|  | medium | 14 | 25 |  |  |
|  | rich | 0 | 1 |  |  |
| Student Origin Distribution |  |  |  | *χ^2^*=2.316 | *p*=0.510 |
|  | Northeast | 0 | 1 |  |  |
|  | East | 9 | 15 |  |  |
|  | Middle | 5 | 3 |  |  |
|  | West | 5 | 6 |  |  |
| Whether Only Child |  |  |  | *χ^2^*=0.016 | *p*=0.900 |
|  | Yes | 8 | 11 |  |  |
|  | No | 11 | 14 |  |  |

Note， ^⁎⁎^*p* <0 .01，After excluding the three subjects with excessive head movement, the significance of the differences between the two groups did not change

**Appendix 4.** **All the differences in functional connections of PFC subregions between the two groups**

| ROI | Brain Regions with the ROI | | voxels | *t* | MNI Coordinates（x，y，z） |
| --- | --- | --- | --- | --- | --- |
|  | Label ID | Gyrus |  |  |  |
| BN3  (Left Superior Frontal Gyrus) | 232 | Right Thalamus | 208 | 5.92 | 12，-2，4 |
|  |  |  |  |  |  |
| BN7  (Left Superior Frontal Gyrus) | 179 | Left Cingulate Gyrus | 179 | 5.00 | -10，24，28 |
|  | 222 | Right Basal Ganglia | 126 | 5.11 | 22，10，8 |
| BN8  (Right Superior Frontal Gyrus) | 3 | Left Superior Frontal Gyrus | 191 | 4.67 | -22，22，62 |
| BN11  (Left Superior Frontal Gyrus) | 64 | Right Precentral Gyrus | 126 | 4.61 | 52，8，14 |
|  |  |  |  |  |  |
| BN12  (Right Superior Frontal Gyrus) | 64 | Right Precentral Gyrus | 232 | 6.30 | 52，8，14 |
| BN16  (Right Middle Frontal Gyrus) | 3 | Left Superior Frontal Gyrus | 123 | 5.13 | -16，20，60 |
| BN20  (Right Middle Frontal Gyrus) | 46 | Right Orbital Gyrus | 199 | 5.66 | 24，50，-18 |
| BN36  (Right Inferior Frontal Gyrus) | 204 | Right Lateral Occipital Cortex | 158 | 5.20 | 12，-100，6 |
| BN38  (Right Inferior Frontal Gyrus) | 3 | Left Superior Frontal Gyrus | 129 | 4.73 | -18，18，60 |
| BN39  (Left Inferior Frontal Gyrus) | 62 | Right Precentral Gyrus | 258 | 5.15 | 46，6，10 |
| BN40  (Right Inferior Frontal Gyrus) | 12 | Right Superior Frontal Gyrus | 231 | 5.88 | 6，42，38 |
|  | 51 | Left Orbital Gyrus | 156 | 4.25 | -42,48,-4 |
| BN42  (Right Orbital Gyrus) | 23 | Left Middle Frontal Gyrus | 119 | 4.63 | -36，34，40 |
| BN44  (Right Orbital Gyrus) | 64 | Right Precentral Gyrus | 154 | 4.50 | 56，12，12 |
| BN46  (Right Orbital Gyrus) | 22 | Right Middle Frontal Gyrus | 236 | 5.69 | 44，56，6 |
|  | 166 | Right Insular Gyrus | 122 | 4.36 | 40，16，-12 |
| BN47  (Left Orbital Gyrus) | 221 | Left Basal Ganglia | 141 | 5.17 | -24，-6，12 |
| BN52  (Right Orbital Gyrus) | 204 | Right Lateral Occipital Cortex | 138 | 4.83 | 12，-100，4 |
| BN65  (Left Paracentral Lobule) | 144 | Right Inferior Parietal Lobule | 382 | 4.95 | 58，-64，36 |

**Appendix 5.**

**Linear regression analysis between the ERQ reappraisal score and the PFC functional connections**

1. The ERQ reappraisal score was significantly negatively correlated with FC: BN3-BN232 (Beta=-0.683, *t* =-3.319，*p =*0.003, 95.0% confidence interval (-0.021，-0.005)).
2. The ERQ reappraisal score was significantly negatively correlated with FC: BN11-BN64 (Beta=-0.577, *t* =-3.140，*p =*0.005, 95.0% confidence interval (-0.032，-0.007)).
3. The ERQ reappraisal score was significantly negatively correlated with FC: BN12-BN64 (Beta=-0.608, *t* =-3.525，*p =*0.002, 95.0% confidence interval (-0.033，-0.008)).
4. The ERQ reappraisal score was significantly negatively correlated with FC: BN12-BN64 (Beta=-0.709, *t* =-3.693，*p =*0.001, 95.0% confidence interval (-0.035，-0.010)).

**Mediation analysis of the ERQ reappraisal score with the PFC functional connections and the BDI-II total score**

**
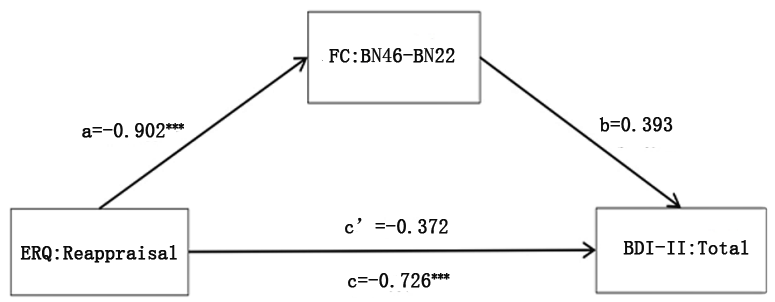
**

Mediation analysis of ERQ: Reappraisal with FC: BN46-BN22 and BDI-II:Total
